# Comparison of Ocular Morphological Parameters Related to Lens Position by Anterior Segment Optical Coherence Tomography and Ultrasound Biomicroscopy

**DOI:** 10.1155/2022/7599631

**Published:** 2022-03-20

**Authors:** Zhiying Yu, Fenglei Wang, Fang Dong, Na Li, Dabo Wang, Ling Wang

**Affiliations:** Department of Ophthalmology, The Affiliated Hospital of Qingdao University, Qingdao, China

## Abstract

**Objective:**

The objective is to compare parameters related to lens position measured using anterior segment optical coherence tomography (AS-OCT) and ultrasound biomicroscopy (UBM) in patients with senile cataract and perform a consistency analysis.

**Methods:**

This prospective study included 102 patients (102 eyes) scheduled for simple cataract surgery. Among the total patients, 44 were men, and 58 were women. AS-OCT (sitting) and UBM (lying) were used to measure the anterior chamber depth (ACD) in horizontal and vertical orientations and the iris-lens contact distance (ILCD) and iris-lens angle (ILA) in inferior, superior, nasal, and temporal quadrants. Paired-sample *t*-test was used to compare ACD, ILCD, and ILA measurements of the two methods, while Pearson's linear correlation and Bland-Altman analyses were used to analyze the correlation and consistency of the two results.

**Results:**

The horizontal (2.499 ± 0.464 mm) and vertical (2.531 ± 0.463 mm) ACD measured using AS-OCT and the horizontal (2.556 ± 0.467 mm) and vertical (2.563 ± 0.479 mm) ACD measured using UBM were significantly different (*P* < 0.001); moreover, the results showed good correlation and agreement. A significant difference was observed between the two methods in terms of ILCD measured in inferior, superior, nasal, and temporal quadrants (*P* < 0.001), and a significant correlation was found between measurements of both methods (*P* < 0.001). Approximately 3.92% (4/102), 0.98% (1/102), 3.92% (4/102), and 2.94% (3/102) of points were outside the 95% limits of agreement in the four quadrants, respectively, and the agreement of the results was good. ILA measured using both methods differed in inferior, superior, nasal, and temporal quadrants (*P*=0.003, 0.011, 0.001, 0.001, respectively), and the correlation was good (*P* < 0.001). The percentage of points outside the 95% limit was higher in inferior, superior, nasal, and temporal quadrants (4.90% (5/102), 5.88% (6/102), 5.88% (6/102), and 6.86% (7/102)) with poor agreement of the results.

**Conclusions:**

The correlation between AS-OCT and UBM in terms of measuring lens position-related parameters was good, but the agreement was unstable. The differences in measurement position (sitting and supine) and/or measurement methods (optics and ultrasound) may lead to variability in results.

## 1. Introduction

Senile cataract is the most common type of cataract, and the disease gradually worsens with age, resulting in an increase in lens thickness and volume. An increase in lens volume may cause the suspensory ligament of the lens to loosen [1]. This may cause shallowing of the anterior chamber and narrowing of the anterior chamber angle, which may subsequently affect aqueous humor circulation, especially during preoperative or intraoperative mydriasis, and even may induce acute angle closure. Therefore, the position of the lens needs to be evaluated in patients with senile cataract. Anterior segment optical coherence tomography (AS-OCT) and ultrasound biomicroscopy (UBM) are commonly used for examining the structure of the anterior segment of the eye based on different optical and ultrasonic principles, respectively. A number of studies have investigated the two methods to examine parameters associated with the anterior chamber angle and compare correlations [2–6]; however, only a few studies have compared parameters related to the lens position using these two methods. In this study, we selected senile cataract patients and measured parameters related to the lens position using AS-OCT and UBM and compared the results of the two methods. Our findings could provide theoretical support for the selection of individualized and safe surgical options for senile cataract patients.

## 2. Data and Methods

### 2.1. Research Participants

This prospective study included 102 patients (102 eyes) who underwent simple cataract surgery in the Affiliated Hospital of Qingdao University from September 2020 to February 2021. All right eyes were examined as the study eyes. The study participants comprised 44 men and 58 women. Patients with a history of ocular surgery; those with poor physical condition, difficulty in the supine position, poor cooperation, or allergies to surface anesthetics; or those whose imaging results could not be quantitatively analyzed owing to poor quality were excluded. This study was conducted after obtaining informed consent from the patients and in accordance with the requirements of the Ethics Committee of the Affiliated Hospital of Qingdao University.

### 2.2. Research Methodology

All participants underwent a detailed ophthalmic examination that included autorefraction (Topcon Ltd, KR-8900, Japan), distance corrected visual acuity (Topcon Ltd, CV-5000, Japan), intraocular pressure measurement (Goldmann applanation tonometer), slit lamp examination (Haag-Streit Ltd, BM 900, Switzerland), and fundus examination (Volk Ltd, VSFNC, USA).

Both AS-OCT (Carl Zeiss Meditec, CIRRUS HD-OCT5000, CA) and UBM (Super Electronic Ltd., SW3200L, China) were performed by the same experienced technician, with AS-OCT measurements performed first, followed by UBM measurements.

AS-OCT examination: under natural lighting, a wide-angle lens was selected, and the anterior chamber scanning mode was used. The patient adopted a sitting position, and the horizontal (nasotemporal) and vertical (inferosuperior) orientations were scanned. Images with good quality were selected and saved, and correlation analysis was performed using the instrument software. Anterior chamber depth (ACD) in two orientations, and iris-lens contact distance (ILCD) and iris-lens angle (ILA) in inferior, superior, nasal, and temporal quadrants were measured separately.

UBM examination: under natural lighting, the patient was placed in the supine position and examined after a drop of surface anesthetic was applied. In the panoramic mode, the anterior chamber images in the horizontal and vertical orientations were measured separately, and images with good quality were saved. ACD in two orientations and ILCD and ILA in four quadrants (12, 6, 3, and 9 o'clock) were measured using the system software.

### 2.3. Measurement Parameters

ACD (mm) is defined as the vertical distance from the inner surface of the central cornea to the anterior surface of the lens.

ILCD (mm) [7] is defined as the distance measured along the iris pigmented epithelium from the pupillary border to the point where the anterior lens surface leaves the iris.

ILA (°) [8]: taking the contact point between the posterior surface of the iris and the anterior surface of the lens as the vertex, a tangent is plotted along this vertex to the posterior surface of the iris and the anterior surface of the lens, respectively, and the resulting angle is the ILA.

**Figure 1:**
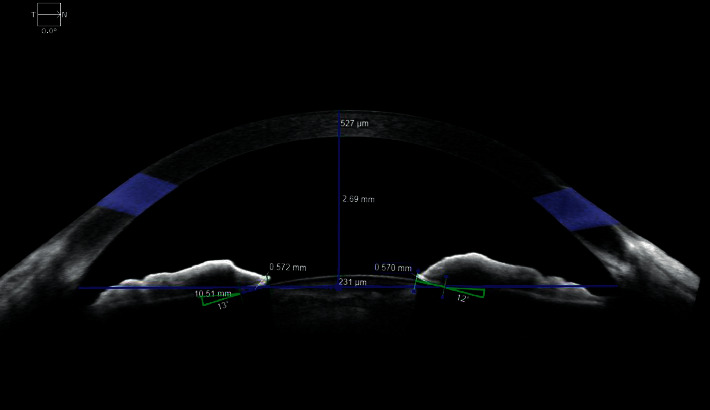
Standardised collection of data from AS-OCT images. ACD = 2.69 mm, ILCD (nasal) = 0.570 mm, ILCD (temporal) = 0.572 mm, ILA (nasal) = 12°, and ILA (temporal) = 13°.

**Figure 2:**
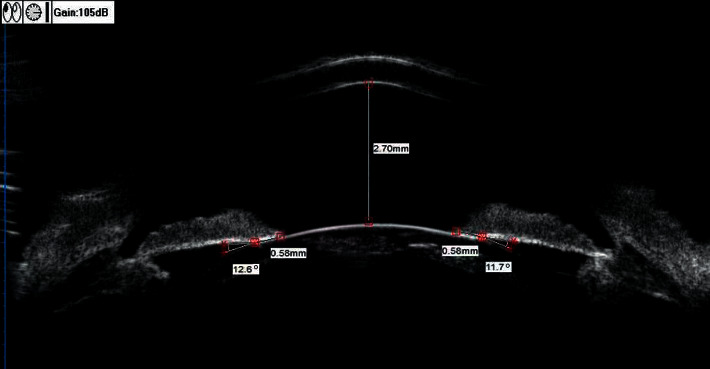
Standardised collection of data from UBM images. ACD = 2.70 mm, ILCD (nasal) = 0.58 mm, ILCD (temporal) = 0.58 mm, ILA(nasal) = 11.7°, and ILA (temporal) = 12.6°.

These parameters are shown in Figures 1 and 2.

### 2.4. Statistical Methods

SPSS 19.0 and Medcalc software were used to analyze data. Quantitative data were tested for normality, and data with a normal distribution are expressed as *x* ± SD. Paired sample *t*-test was used to compare ACD, ILCD, and ILA measurements of the two instruments. Pearson's linear correlation analysis was used to determine the correlation between the two results, and Bland-Altman analysis was used to analyze the agreement of the two results and calculate 95% limits of agreement (LoA). A *P*-value of <0.05 was considered significant.

## 3. Results

### 3.1. Comparison and Correlation of the Two Measurement Methods

Differences between results of ACD measured using AS-OCT and UBM in both horizontal orientation (180°) and vertical orientation (90°) were significant, and both showed good correlation (Table 1).

The differences in ILCD and ILA measured using AS-OCT and UBM were significant for superior, inferior, nasal, and temporal ILCD and ILA, and a positive correlation was found between the measurements of both the methods (Table 2).

### 3.2. AS-OCT and UBM Measurement Agreement Analysis of Each Parameter

**Figure 3:**
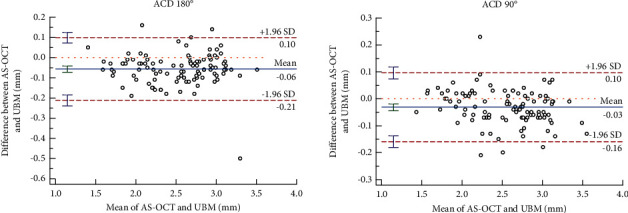
Agreements of ACD measured by AS-OCT and UBM (Bland–Altman diagram).

Bland-Altman analysis showed good agreement between ACD values measured using the two methods. The 95% agreement intervals were −0.072 to 0.041 and −0.044 to 0.018, respectively, and the proportions of out-of-line points were 2.94% (3/102) and 3.92% (4/102), respectively, with a narrower 95% LoA range of −0.21 to 0.10 mm in the Bland-Altman analysis (Figure 3).

**Figure 4:**
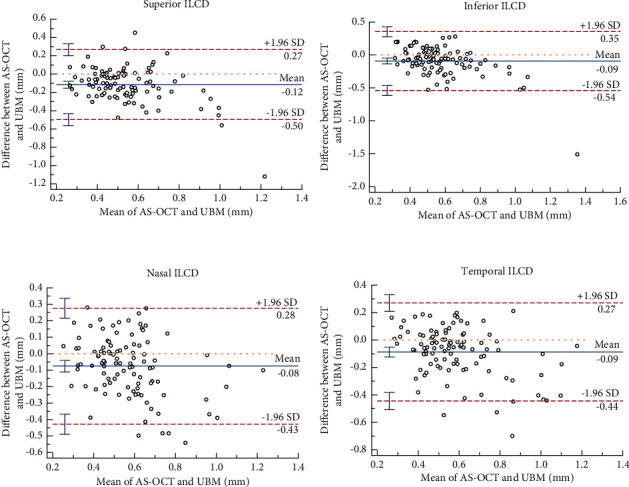
Agreements of ILCD measured by AS-OCT and UBM (Bland–Altman diagram).

Bland-Altman analysis showed that 3.92% (4/102), 0.98% (1/102), 3.92% (4/102), and 2.94% (3/102) of ILCD points in the inferior, superior, nasal, and temporal quadrants were outside the 95% agreement limits, while a 95% LoA range of −0.54 to 0.35 mm was narrower; hence, the results of these two parameters were considered to be in good agreement (Figure 4).

**Figure 5:**
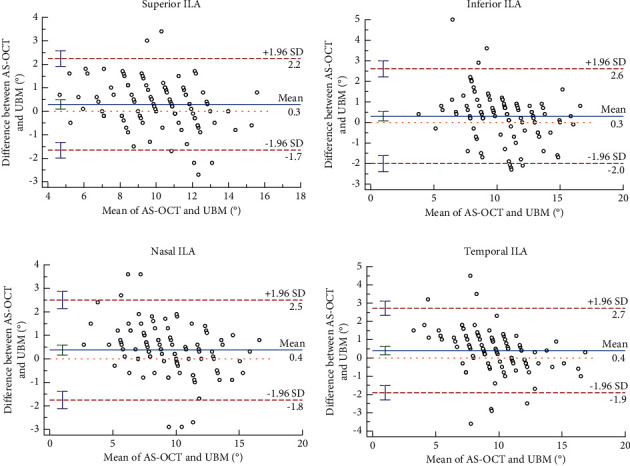
Agreements of ILA measured by AS-OCT and UBM (Bland–Altman diagram).

Bland-Altman analysis showed that the ILA results were higher in the inferior, superior, nasal, and temporal quadrants with a high percentage of points outside the 95% line: 4.90% (5/102), 5.88% (6/102), 5.88% (6/102), and 6.86% (7/102). Only points greater than 95% were within the limits of agreement in the superior quadrant. The 95% LoA range of −2.0 to 2.7 was analyzed, and the results showed that the ILA in the four quadrants in various maxima was 2.25, 4.47, 3.39, and 2.72, respectively, and this difference was considered to be large when taken together; hence, the consistency of both methods was regarded as poor (Figure 5).

## 4. Discussion

We observed varying degrees of intraoperative lens position abnormalities in some senile cataract patients, with the exception of significant lens subluxation and lens luxation, which are mainly because of anterior displacement of the lens-iris diaphragm caused by increased lens thickness or/and relaxation of the suspensory ligament of the lens. If this abnormality in lens position is not evaluated prior to surgery, it may be induced to acute angle closure by preoperative or intraoperative mydriasis, intraoperative dissociation of the suspensory ligament of the lens, and persistent postoperative high intraocular pressure. Therefore, preoperative evaluation of the lens position has attracted more and more attention from clinicians.

Owing to the deep location of the suspensory ligament of the lens, its fibrous distribution, the varying sparseness of each quadrant, and the often variable attachment position of the lens and ciliary process, lens imaging (AS-OCT and UBM) cannot clearly visualize the morphology of the suspensory ligament and the extent of dissociation in all quadrants. We can only indirectly obtain the lens position from the equator of the lens to the ciliary process [9–11]. In our imaging study of UBM with lens subluxation, we found that when relaxation or dissociation of the suspensory ligament occurs, the lens is displaced toward the ciliary body and iris root in the quadrant on the contralateral suspensory ligament, the contact area between the anterior surface of the lens and the posterior surface of the iris increases (ILCD becomes longer), the iris-lens angle disappears (ILA becomes smaller), and there is a significant negative correlation between ILA and ILCD [12]. Since these two markers can be used as sensitive indicators for evaluating lens subluxation, we need to assess if they can be combined with AS-OCT to assess the relative position of the lens in patients with senile cataract. The two different measurements complement each other to comprehensively assess the relative position of the lens in patients with senile cataract.

The lens thickens and the anterior chamber become shallow as age increases [13, 14]. However, ACD continues to become more shallow with increasing age without an increase in lens thickness, suggesting that lens thickness is not the only factor that causes the anterior chamber to appear more shallow; this finding suggests that the anterior chamber becomes shallow owing to the relaxation of the suspensory ligament of the lens, which displaces the lens forward [15–18]. Our findings revealed that the mean values of ACD measured using AS-OCT in the horizontal and vertical orientations were 2.499 ± 0.464 mm and 2.531 ± 0.463 mm, respectively, in the sitting position, and the mean values of UBM in the supine position were 2.556 ± 0.467 mm and 2.563 ± 0.479 mm in both orientations, respectively; moreover, ACD in both orientations measured using UBM was greater than that measured using AS-OCT, which may be related to the slightly receding lens-iris diaphragm in the supine position and the increased distance from the posterior surface of the central cornea. This finding is consistent with the results of the analysis of the Chinese study conducted by Shen et al. [19]. The deepening of ACD in the supine position corresponds to an increase in ILCD and a decrease in ILA in four quadrants in the supine position. These results all suggest that suspensory ligament relaxation may be present in senile cataract patients included in this study. It was not possible to distinguish whether this difference was because of the measurement method, body position, or both.

UBM can clearly display images of the anterior and part of the middle segments of the eye and measure each biological parameter [20]. It clearly displayed the different tissues of the anterior segment in high resolution and dynamics. The results obtained were independent of refractive media and were found to be helpful in quantitatively measuring relevant parameters [21]. In contrast, the imaging methods used in this study have several disadvantages. The UBM probe requires a water bath to contact the cornea, it should be performed with the patient in a supine position, the examination can cause mechanical damage such as corneal epithelial defects due to improper handling, and infection may occur after the examination [22, 23]. OCT is an optical diagnostic technique that was developed in the 1990s [24–26]. It is a noninvasive noncontact procedure, has high safety, can be performed independent of the degree of corneal opacification, and can clearly display some of the anterior chamber angle structures such as scleral spur [27]. In contrast, it has some disadvantages. It cannot clearly display the posterior iris structures owing to the effects of the iris pigment epithelium and refractive media. Therefore, both methods can be used for routine measurement of the anterior chamber. In patients who cannot lie supine, have poor cooperation, or have a corneal injury or infectious disease and other conditions that make UBM examination unsuitable, AS-OCT is preferred for measuring the anterior segment. In patients who have significant clouding of the refractive media and require observation of the morphological structure of the posterior chamber, ciliary body, and anterior suspensory ligament, UBM is preferred for examination. Cui et al. [28] used UBM and OCT to examine the agreement of the anterior chamber angle; the anterior chamber angle results obtained using both methods had a good agreement when performed in patients with significant closure and wide anterior chamber angle but had slightly less agreement when performed in those with a narrow anterior chamber angle. Yu et al. [2] measured the anterior chamber angle-related parameters using AS-OCT and UBM and found no significant differences between the two methods and good correlation and agreement between them. However, different findings were obtained. Wang et al. [29] showed poor agreement between high-resolution AS-OCT and UBM for measuring narrow anterior chamber angle in a study of patients with closed-angle glaucoma. At present, a few comparative studies have been conducted to compare the two devices for measuring ILCD and ILA. The results of the study conducted by Mansoori and Balakrishna [7], who evaluated changes in ocular morphology in patients with primary angle-closure glaucoma after laser peripheral iridotomy using UBM, showed that ILCD was greater in PAGG patients than in normal participants, while ILA was smaller in PAGG patients than in normal participants.

In the agreement analysis of the two measurement methods, we found that points greater than 95% of the results of the ACD measurement obtained using AS-OCT and UBM were within the limits of agreement and in good agreement. Significant differences were observed between ILCDs measured using both the methods in the four quadrants; approximately 3.92% (4/102), 0.98% (1/102), 3.92% (4/102), and 2.94% (3/102) of points were outside the 95% agreement limits in the inferior, superior, nasal, and temporal quadrants, respectively, and the agreement of the results was good. Moreover, differences were observed in the ILA measured by both methods in the four quadrants, with a higher percentage of points outside the 95% limit in the inferior, superior, nasal, and temporal quadrants: 4.90% (5/102), 5.88% (6/102), 5.88% (6/102), and 6.86% (7/102), with poor agreement of the results. This may be due to poor imaging of the posterior iris tissue using AS-OCT. Although AS-OCT is more convenient, it is constrained by its optical examination limitations and cannot show posterior iris structures. As an acoustic examination that is independent of refractive media, UBM allows observation of the posterior iris ciliary body and compensates for the shortcomings of AS-OCT. When these two imaging methods and image analysis are performed, although we used the same point for comparison whenever possible, it is difficult to achieve the exact agreement among the four quadrants, and slight changes in measurement position may cause variability in the results. In addition, when a patient switches his position from sitting to supine, there will be a slight rotation of the eye, which may increase the deviation of the measurement points of the two methods. This is the limitation of our study. In the follow-up study, we will look forward to a navigation system in the examination to ensure the consistency of measuring points when the body position changes.

In conclusion, in senile cataract patients, although significant differences were found between AS-OCT and UBM in measuring parameters related to ACD and lens position, the correlation between the two is good, but the agreement of some parameters is unstable. Hence, these two methods complement each other and can be used to preoperatively evaluate the relative position of the lens in patients with senile cataract and provide a theoretical basis for selecting the appropriate treatment plan for them.

## Figures and Tables

**Table 1:** Pearson correlation analysis and *T*-test of paired samples of horizontal and vertical ACD using AS-OCT and UBM.

	AS-OCT	UBM	*T*-test		Pearson correlation	
	Mean ± SD	Mean ± SD	*t*	*P*	*r*	*P*
ACD (horizontal) (mm)	2.499 ± 0.464	2.556 ± 0.467	−7.191	0.001^*∗*^	0.986	0.001^*∗*^
ACD (vertical) (mm)	2.531 ± 0.463	2.563 ± 0.479	−4.847	0.001^*∗*^	0.991	0.001^*∗*^

SD means standard deviation; ^*∗*^*P* < 0.001.

**Table 2:** Pearson Correlation analysis and *T*-test of paired samples of ILCD and ILA using AS-OCT and UBM.

	AS-OCT	UBM	*T*-test		Pearson correlation	
	Mean ± SD	Mean ± SD	*t*	*P*	*r*	*P*
ILCD (mm)						
Superior	0.480 ± 0.156	0.595 ± 0.234	−5.569	0.001^*∗*^	0.561	0.001^*∗*^
Inferior	0.523 ± 0.145	0.616 ± 0.260	−4.144	0.001^*∗*^	0.484	0.001^*∗*^
Nasal	0.532 ± 0.168	0.607 ± 0.223	−4.242	0.001^*∗*^	0.611	0.001^*∗*^
Temporal	0.544 ± 0.175	0.630 ± 0.244	−4.821	0.001^*∗*^	0.667	0.001^*∗*^
ILA (°)						
Superior	10.275 ± 2.167	9.979 ± 2.437	2.991	0.003	0.913	0.001^*∗*^
Inferior	10.578 ± 2.479	10.277 ± 2.821	2.587	0.011	0.909	0.001^*∗*^
Nasal	9.647 ± 2.720	9.270 ± 3.089	3.509	0.001	0.938	0.001^*∗*^
Temporal	9.804 ± 2.421	9.397 ± 2.894	3.484	0.001	0.917	0.001^*∗*^

SD means standard deviation; ^*∗*^*P* < 0.001.

## Data Availability

The data used to support the findings of this study are available from the corresponding author upon request.

## References

[1] Lowe R. F. (1970). Anterior lens displacement with age. *British Journal of Ophthalmology*.

[2] Yu Z. Y., Huang T., Lu L., Qu B. (2020). Comparison of measurements of anterior chamber angle via anterior segment optical coherence tomography and ultrasound biomicroscopy. *World Journal of Clinical Cases*.

[3] Ma X. Y., Zhu D., Zou J., Zhang W. J., Cao Y. L. (2016). Comparison of ultrasound biomicroscopy and spectraldomain anterior segment optical coherence tomography in evaluation of anterior segment after laser peripheral iridotomy. *International Journal of Ophthalmology*.

[4] Mansouri K., Sommerhalder J., Shaarawy T. (2010). Prospective comparison of ultrasound biomicroscopy and anterior segment optical coherence tomography for evaluation of anterior chamber dimensions in European eyes with primary angle closure. *Eye*.

[5] Dada T., Sihota R., Gadia R., Aggarwal A., Mandal S., Gupta V. (2007). Comparison of anterior segment optical coherence tomography and ultrasound biomicroscopy for assessment of the anterior segment. *Journal of Cataract and Refractive Surgery*.

[6] Li X. Y., Chang P. J., Li Z. L. (2020). Agreement between anterior segment parameters obtained by a new ultrasound biomicroscopy and a swept-source fourier-domain anterior segment optical coherence tomography. *Expert Review of Medical Devices*.

[7] Mansoori T., Balakrishna N. (2017). Anterior segment morphology in primary angle closure glaucoma using ultrasound biomicroscopy. *Journal of Current Glaucoma Practice*.

[8] Teekhasaenee C., Ritch R. (1999). Combined phacoemulsiﬁcation and goniosynechialysis for uncontrolled chronic angleclosure glaucoma after acute angle-closure glaucoma. *Ophthalmology*.

[9] See J. L. (2009). Imaging of the anterior segment in glaucoma. *Clinical and Experimental Ophthalmology*.

[10] Liu Y. Z., Liu Y. H., Wu M. X. (2004). Clinical applications of ultrasound biomicroscopy in diagnosis and treatment of lens subluxation. *Chinese Journal of Ophthalmology*.

[11] McWhae J. A., Crichton A. C., Rinke M (2003). Ultrasound biomicroscopy for the assessment of zonules after ocular trauma. *Ophthalmology*.

[12] Wang F., Wang D., Wang L. (2019). Characteristic manifestations regarding ultrasound biomicroscopy morphological data in the diagnosis of acute angle closure secondary to lens subluxation. *BioMed Reseach International*.

[13] Roters S., Hellmich M., Szurman P. (2002). Prediction of axial length on the basis of vitreous body length and lens thickness. *Journal of Cataract and Refractive Surgery*.

[14] Allouch C., Touzeau O., Kopito R., Borderie V., Laroche L. (2005). Crystalline lens biometry using A-scan ultrasound and the orbscan device. *Journal Francais D Ophtalmologie*.

[15] Yong K. L., Gong T., Nongpiur M. E. (2014). Myopia in asian sub jects with primary angle closure: implications for glaucoma trends in East Asia. *Ophthalmology*.

[16] Barkana Y., Shihadeh W., Oliveira C., Tello C., Liebmann J. M., Ritch R. (2006). Angle closure in highly myopic eyes. *Ophthalmology*.

[17] Ritch R. (1994). Exfoliation syndrome and oceludable angles. *Transactions American Ophthalmological Society*.

[18] Lim A. S. M. (2004). *Lowe-Lim Primary Closed Angle Glaucoma*.

[19] Shen L., Wang X. N., Li D. J. (2018). Comparison of swept source anterior segment optical coherence tomography and ultrasound biomicroscopy in measurement of anterior chamber depth and anterior chamber angle data in age-related cataract patients. *Chinese Journal of Ophthalmology*.

[20] Torrfs V., Allemann N., Erwenn C. M. (2005). Ultrasound biomicroscopy features of iris and ciliary body melanomas before and after brachytherapy. *Ophthalmic Surgery Lasers Imaging Retina*.

[21] Konstantopoulos A., Hossain P., Anderson D. F. (2007). Recent advances in ophthalmic anterior segment imaging :a new era for ophthalmic diagnosis?. *British Journal of Ophthalmology*.

[22] Meinhardt B., Stachs O., Stave J., Beck R., Guthoff R. (2006). Evaluation of biometric methods for measuring the anterior chamber depth in the non-contact mode. *Graefes Archive for Clinical and Experimental Ophthalmology*.

[23] Silverman R. H. (2016). Focused ultrasound in ophthalmology. *Clinical Ophthalmology*.

[24] Hoerauf H., Wirbelauer C., Scholz C. (2000). Slit-lamp- adapted optical coherence tomography of the anterior segment. *Graefes Archive for Clinical and Experimental Ophthalmology*.

[25] Baikoff G., Lutun E., Ferraz C., Wei J. (2004). Static and dynamic analysis of the anterior segment with optical coherence tomography. *Journal of Cataract and Refractive Surgery*.

[26] Nemeth G., Vajas A., Tsorbatzoglou A., Kolozsvari B., Modis L., Berta A. (2007). Assessment and reproducibility of anterior chamberdepth measurement with anterior segment opticalcoherence tomography compared with immersion ultrasonography. *Journal of Cataract and Refractive Surgery*.

[27] Radhakrtshnan S., Huang D. (2005). Optical coherence tomography imaging of the anterior chamber angle. *Ophthalmology Clinics of North America*.

[28] Cui L. J., Huang L. B., Xu G. X. (2016). Anterior chamber angle examinations using UBM versus OCT. *Journal of Clinical Ophthalmology*.

[29] Wang D., Pekmezci M., Basham R. P., He M., Seider M. I., Lin S. C. (2009). Comparison of different modes in optical coherence tomography and ultrasound biomicroscopy in anterior chamber angle assessment. *Journal of Glaucoma*.

